# Nature of the Dielectric Anomaly in Na_0.5_Bi_0.5_TiO_3_–Based Ferrolectrics

**DOI:** 10.3390/ma18235289

**Published:** 2025-11-24

**Authors:** Eriks Birks, Marija Dunce, Šarūnas Svirskas, Algimantas Kežionis, Juras Banys, Andrei Kholkin

**Affiliations:** 1 Institute of Solid State Physics, University of Latvia, Kengaraga 8, LV-1063 Riga, Latvia; eriks.birks@cfi.lu.lv (E.B.); marija.dunce@cfi.lu.lv (M.D.); 2 Faculty of Physics, Vilnius University, Saulėtekio al. 3, LT-10257 Vilnius, Lithuania; sarunas.svirkas@ff.vu.lt (Š.S.); algimantas.kezionis@ff.vu.lt (A.K.); juras.banys@ff.vu.lt (J.B.)

**Keywords:** ferroelectric relaxors, phase transition, dielectric anomaly

## Abstract

The temperature–frequency dependence of dielectric permittivity in Na_0.5_Bi_0.5_TiO_3_ (NBT) -based compositions displays a diffused, frequency-independent maximum along with a frequency-dependent shoulder below this maximum. This behavior deviates from that of both classical ferroelectrics and conventional relaxor ferroelectrics, and its interpretation is further complicated by challenges in linking it to known structural phase transitions. This study proposes a new interpretation of the dielectric behavior of NBT-based materials through a comparative analysis of temperature–frequency permittivity data in both unpoled and poled NBT samples and 0.95Na_0.5_Bi_0.5_TiO_3_–0.05CaTiO_3_ solid solution over a broad frequency range (10 Hz–100 MHz). Results reveal that the steep permittivity change between the maximum and shoulder—accompanied by pronounced thermal hysteresis—can be attributed to a phase transition between two non-ferroelectric phases. When this contribution is excluded, the dielectric response aligns with classical relaxor ferroelectric behavior. To reconcile this with other known properties of NBT, the “breathing” model is employed, offering a unified framework for understanding its relaxor-like characteristics.

## 1. Introduction

At the moment, lead-based perovskite ferroelectrics dominate this market segment, but their use is limited due to health and environmental issues [1]. NBT-based compositions are among the most promising lead-free substitutes here. Structure and phase transitions of these materials are among the aspects being in focus of the research. While the poled state of NBT at room temperature is undoubtedly ferroelectric, with R3c symmetry [2], the unpoled state does not show presence of domains or any other signs of ferroelectric state [3]. In this respect, the unpoled state and the poled state have to be considered separately. Symmetry in the unpoled state is an issue that has been debated for a long time. Seemingly, X-ray and neutron diffraction patterns satisfactorily conform to the R3c symmetry [4,5,6]. However, the observed complex shape of X-ray diffraction peaks [7,8,9,10,11], as well as simultaneous presence of superlattice reflections belonging to different symmetries [11,12] indicate a coexistence of the R3c symmetry phase with some other phase. As a result, coexistence of phases of R3c and Cc [9], R3c and Pm3-m [8], R3c and 4Pbm [13,14], as well as R3c and Pnma [10] symmetries was suggested. Unfortunately, distortions of the NBT pseudo-cubic unit cell are small, and the comparison of various coexisting phases yields similar quantitative results [10,15]. More sophisticated approaches [16,17,18], paying attention to differences between the local and global symmetry in the presence of specific mesoscopic structures, extend the range of possible interpretations.

In the temperature region above 300 °C, interpretation of NBT crystalline structure is more convincing: a transition to a phase with weak tetragonal P4bm symmetry takes place through the phase coexistence region at 300–320 °C (this region could be even wider) [19,20]. In all reported studies, a coexistence region with phase of R3c symmetry was found. Further, at 500–540 °C, this phase transforms into a cubic phase of Pm3-m symmetry [4].

Characterization of crystalline structures below the phase transition to the tetragonal phase is problematic. Abandoning the idea of pure R3c phase at room temperature has motivated researchers to perform more detailed studies of structure upon increasing of temperature in the range where existence of pure R3c phase was assumed earlier—up to the temperature of the phase transition to the tetragonal phase [4]. Thus, when analyzing neutron elastic scattering measurements, short-range rhombohedral (R3c) and tetragonal (P4bm) correlations were detected in the temperature range 200–300 °C [21]. While, according to TEM studies, at around 200 °C, orthorhombic sheets of Pnma symmetry appear, separating areas of R3c symmetry [16]. In such modulated mesoscopic structures, dipole moments in neighboring R3c areas should be oriented in opposite directions, creating an association with the antiferroelectric state. Upon increasing temperature, concentration of the sheets increases until, at 280 °C, a transfer to complete orthorhombic phase takes place. This phase exists in a narrow temperature range, before the phase transition to the tetragonal phase occurs at 320 °C [12]. Unfortunately, such an interpretation has not received better experimental confirmation.

The problems with identification of structure and especially the diffused changes in it dependent on temperature do not allow for a convincing connection with the anomalies observed in physical properties, first of all, dielectric permittivity. In general, the diffused peak of the temperature dependence of dielectric permittivity ε′(T), which in NBT is located at a temperature around 330–340 °C, usually denoted as T_c_, is almost independent of frequency. (It is important to stress that T_c_ here may not be a ferroelectric phase transition temperature.) However, there are some exemptions. Specifically, in NBT-PbZrO_3_ solid solutions, a clear relaxor-characteristic dependence of ε′(T) on frequency is observed in the region of T_c_ [22]. In addition, contribution from DC conductivity, which is dependent on the composition and increases with increasing temperature, can also create frequency-dependent contributions in the region of T_c_ [23].

It should be noted that the dielectric permittivity peak located at T_c_ is strongly asymmetric—it has a steep part in the low-temperature side and a diffused part in the high-temperature side. In ferroelectrics, a ε′(T) peak usually indicates a ferroelectric phase transition. Therefore, there were attempts to apply the Curie–Weiss law, which is valid above T_c_ for traditional ferroelectrics, and extract its parameters also in NBT [21,24,25]. However, such an approach does not seem to be plausible in this case, as it is substantiated only for a ferroelectric–paraelectric or antiferroelectric–paraelectric phase transition. In NBT, the ferroelectric state exists only after poling and, even then, it is destroyed upon heating at the depolarization temperature T_d_ = 180 °C, significantly below T_c_. Moreover, the Curie–Weiss law in NBT is always applied in a temperature range starting from a temperature significantly above T_c_—which is not well grounded, even conceptually.

Sometimes, NBT is assumed to be in an antiferroelectric state within the temperature range between the region of dielectric permittivity shoulder and T_c_ [26,27,28]. Nevertheless, there is no evidence in experimental studies of the symmetry of the crystalline structure confirming this hypothesis [4,12,16,21], whereas the features of double polarization hysteresis loops sometimes observed in NBT [23,29] are evidence of any kind of the first-order phase transition, including a relaxor–ferroelectric one, and do not necessarily indicate the presence of an antiferroelectric phase. Therefore, there is not sufficient motivation to insist on the relation with the antiferroelectric phase, and the peak at T_c_ could be caused by some other reason.

In the above-considered studies, the dielectric permittivity peak at T_c_ in NBT-based compositions was attributed to a phase transition. In contrast, Ge et al. have found that the temperature at which tetragonal splitting appears in XRD patterns upon increasing of temperature coincides with the position of the low-temperature steep part—jump/drop—of this peak in NBT [21]. Based on the detected anomalies of elastic properties, Hiruma et al. [30] have also assumed the existence of a rhombohedral–tetragonal phase transition in the region of this jump/drop. It follows from their report, but they did not pay attention to this correlation.

Diaz et al. have jointly analyzed temperature dependence of dielectric permittivity, Young’s modulus, and thermally stimulated depolarization currents in NBT ceramics [31]. The authors have managed to describe temperature dependences of the studied properties, including dielectric permittivity at one particular frequency (1 MHz) in the temperature region starting slightly below the T_c_ and extending to the high-temperature range as the result of accumulation of free charges at grain boundaries due to variations in conductivity between grains and grain boundaries. Certainly, increased conductivity at elevated temperatures plays an essential role in electrical properties [32]. However, the offered model is based purely on an RC equivalent circuit. This means that, in the framework of this model, dielectric properties are of Debye type, which does not correspond to the experimentally observed nature of the dielectric permittivity peak. It is also not quite clear how to adopt this model for single crystals, where dielectric properties are similar [33,34].

A common feature distinguishing NBT-based compositions from other ferroelectrics is the existence of a so-called shoulder in dielectric permittivity temperature dependence below the diffused peak at T_c_. Contrary to the peak, which is frequency-independent, this shoulder has a well-expressed frequency dependence, resembling the behavior of relaxor ferroelectrics. In spite of the clearly observable frequency dependence, this shoulder is sometimes associated with depolarization. The latter stems from the fact that the location of the shoulder coincides with the temperature range where the poled state is destroyed upon heating. Due to this, the dielectric permittivity shoulder was used to identify the depolarization temperature T_d_ in NBT-based compositions instead of determining it from heating experiments of poled samples [31,35,36,37]. However, such a coincidence, if it is not substantiated by other evidence of a phase transition (the term “depolarization” in the unpoled state is not applicable), appears to be accidental. First of all, the jump in dielectric permittivity dependence observed in poled NBT-based ferroelectrics, in fact, is related to the destruction of a ferroelectric domain state. Therefore, there is no reason to address the shoulder appearing upon heating in the unpoled NBT-based samples in the same manner as this jump if there is no evidence of ferroelectric domains. Such is the case of NBT [3]. In addition, there are NBT-based compositions in which destruction of the poled state, depolarization, occurs at temperatures clearly below the temperature range of the shoulder [38,39].

The fact that dispersion of dielectric permittivity in the temperature range of the shoulder resembles relaxor ferroelectrics, as well as the above mentioned hypothesis that antiferroelectric state is present in the temperature range between the shoulder and the peak, has inspired the idea of antiferroelectric nanoregions embedded in nonpolar matrix in (1−x)NBT-xBaTiO_3_ solid solutions in the concentration range of the morphotropic phase boundary [40,41]. These nanoregions were assumed to be responsible for the observed frequency-dependent dielectric permittivity shoulder. Meanwhile, after detecting the simultaneous presence of superlattice reflections attributed to phases of R3c and P4bm symmetry by TEM in 0.95NBT-0.06BaTiO_3_, Jo et al. have assumed the existence of two kinds of polar nanoregions (PNRs) with different crystalline symmetry—rhombohedral and tetragonal [42]. The shoulder mainly contains contributions from relaxation of the rhombohedral PNRs, whereas the peak of dielectric permittivity at T_c_ is related to contribution from a specific combination of two processes: transformation of the rhombohedral PNRs to the tetragonal ones and their relaxation. Taking into account later comprehensive studies of the crystalline structure of NBT [8,9,10,13,14], which have confirmed the presence of a polar R3c phase among several coexisting phases, at least a part of this concept—related to the PNRs of rhombohedral symmetry—appears to be well substantiated.

Recently, Fan et al. have focused their studies on the dielectric permittivity shoulder after different treatments (rapid cooling, cooling at applied electric field, and mechanical stress) [43]. They have found that the applied treatment has an impact on the shoulder, leaving the part of dielectric permittivity dependence below it practically without changes. The authors have explained the observed features using a so-called “breathing” model, where movement of PNR domain walls pinned by defects is considered to be the reason of the dielectric dispersion, instead of changes in the polarization direction of PNRs [44].

Despite the fact that the frequency dependence of dielectric properties of NBT-based compositions is well documented, only a few studies were focused on deeper analysis of the dielectric dispersion. Besides the above-mentioned work of Jo et al. [42], we would like to pay attention to the research by Petzelt et al. [45]. In this work, the authors have extracted several relaxation mechanisms, extending below and above T_c_, from measurements of temperature dependence of the high-frequency dielectric dispersion in NBT. Remarkably, the mechanism located in the range of the longest relaxation times had quite a smooth relaxation time temperature dependence with values ranging from 10–11 at 900 K down to 10^−8^ at 100 K, derived using the Cole–Cole formula on the dielectric spectra. This dependence does not reveal any signs of freezing. However, dielectric strength has a well-expressed peak at T_c_ with a shape resembling the dielectric permittivity peak. Similar results were obtained when studying the frequency dispersion in 0.7NBT-0.3Sr_0.2_Bi_0.7_TiO_3_ solid solution, which also possesses the temperature dependence of dielectric permittivity characteristic of NBT [46]. Again, the application of the Cole–Cole formula to the dielectric dispersion did not reveal any anomalies in temperature dependence of the extracted relaxation time. At the same time, temperature dependence of dielectric permittivity around T_c_, including even the minimum observed between the peak at T_c_ and the shoulder, was concluded to be determined solely by static dielectric permittivity. It should be noted that dielectric dispersion in 0.7NBT-0.3Sr_0.2_Bi_0.7_TiO_3_ at the considered temperatures is apparently limited to the frequency range from the GHz region and to the highest measurement frequencies [34,46].

It is important to note that the steep part of ε′(T) peak has revealed the thermal hysteresis. Recently, based on comparison of NBT with 0.95NBT-0.05CaTiO_3_, where the steep change in ε′(T) even resembles a jump and obviously corresponds to the phase transition between orthorhombic and tetragonal phases, we have made an assumption that such an interpretation can be extended also to NBT [10]. Namely, the steep change in ε′(T), possessing thermal hysteresis, corresponds to the first-order phase transition between the low-temperature state of unclear symmetry or rather coexisting phases and the high-temperature tetragonal phase. This conclusion corresponds well to the results reported by Ge et al. [21]. In comparison with 0.95NBT-0.05CaTiO_3_, the phase transition in NBT is more diffused. We have compared anomalies of several physical properties at this phase transition for both compositions [47]. One of our significant observations was that temperature dependence of Young’s modulus, which is acknowledged as a sensitive tool for detecting phase transitions, reveals a clear anomaly close to the phase transition to the tetragonal phase with thermal hysteresis, corresponding to the hysteresis of dielectric permittivity. In the present study, dielectric dispersion is compared for the unpoled and poled NBT and 0.95NBT-0.05CaTiO_3_ ceramics. A new interpretation is suggested for temperature dependence of dielectric permittivity in NBT-based solid solutions in relation with the non-ferroelectric phase transition.

## 2. Materials and Methods

Na_0.5_Bi_0.5_TiO_3_ (NBT) and 0.95Na_0.5_Bi_0.5_TiO_3_–0.05CaTiO_3_ (0.95NBT–0.05CT) ceramics were synthesized by the conventional solid-state reaction method with a two-stage calcination, as described in detail in our previous work [10]. Chemical-grade Na_2_CO_3_, Bi_2_O_3_, CaCO_3_, and TiO_2_ powders (purity > 99.5%) were used as starting materials. The mixed and milled powders were calcined first at 850 °C and then at 1000 °C, followed by sintering at 1160 °C for NBT and at 1180 °C for 0.95NBT-0.05CT for 2 h. To minimize Bi evaporation, calcination and sintering were carried out in covered platinum crucibles with the samples embedded in powders of the same composition.

The sintered ceramics were cut and polished to a thickness of approximately 0.3 mm, and the plates were electroded by applying a silver paste and firing it at 400 °C for further examination. In the present work, dielectric permittivity measurements were performed for the unpoled and poled NBT and 0.95NBT-0.05CT ceramics. Poling of both ceramics was performed by applying an electric field of 70 kV/cm at room temperature.

The broadband dielectric spectroscopy measurements were performed using a custom-made ultrabroadband spectrometer. In this spectrometer, the sample is placed in a coaxial line constructed from platinum-coated ceramic tubes. This design allows heating of the coaxial line to high temperatures (up to 800 °C or higher). Heating is achieved by applying a DC current to a wire wrapped around the outer shell of the coaxial line. This part of the sample compartment is well isolated from the environment. A K-type thermocouple, positioned inside the inner tube of the coaxial line, measures the temperature at the point where the sample surface contacts the inner tube. Measurements in this study were taken in 2 K increments, with the sample temperature stabilized at each step.

Coverage of the 10 Hz–100 MHz frequency range was achieved by combining two experimental methods. An electrical relay was used to switch between the two setups, enabling simultaneous measurement of the entire spectrum.

The low-frequency method (10 Hz–10 MHz) employed a TiePie Handyscope HS3 oscilloscope (TiePie Engineering, Sneek, The Netherlands) with a built-in signal generator. It generates sinusoidal waves and measures the current flowing through the sample. A detailed schematic of this method is provided in the literature [48].

The higher frequencies (10–100 MHz) were covered by using vector network analyzer Agilent E5071C (Keysight Technologies, Santa Clara, CA, USA). In this setup, the complex reflection and transmission coefficient are measured. The full description of the experimental technique can be found in the literature [49].

X-ray diffraction (XRD) patterns and scanning electron microscopy (SEM) micrographs were also obtained to confirm that the structural and microstructural properties of the samples are consistent with our previous studies, indicating a single-phase composition and a well-developed microstructure. Detailed analyses of the structure, microstructure, and other physical properties can be found in our earlier publications [10,47].

## 3. Results

**Figure 1:**
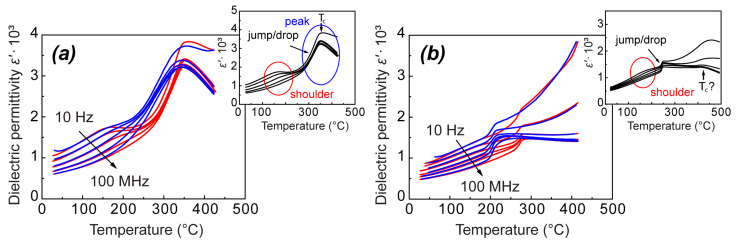
Temperature dependences of the real part of dielectric permittivity ε′(T) for the unpoled NBT (**a**) and 0.95NBT-0.05CT (**b**) samples at several frequencies upon heating (red lines) and followed cooling (blue lines), grasping the most characteristic features of NBT-based solid solutions. The additional graphs represent a schematic view of these dependences denoting the characteristic anomalies. The additional graph for 0.95NBT-0.05CT is presented from our previous study [10], covering a wider measurement temperature range.

In general, temperature–frequency dependences of the real part of dielectric permittivity ε′(T, f) for both NBT and 0.95NBT-0.05CT in the unpoled state resemble the behavior characteristic of the whole family of NBT-based compositions, as depicted in Figure 1. They possess a frequency-dependent shoulder at lower temperatures and a frequency-independent anomaly at higher temperatures. In the case of NBT, the frequency-independent anomaly is represented by a diffused peak with a maximum at a temperature T_c_ and a steep change in its low-temperature part (Figure 1a), which corresponds well to the literature. Further, we will denote this steep change as a diffused jump/drop upon increasing and decreasing of temperature, respectively. Whereas, in the case of 0.95NBT-0.05CT, it seems that solely the steep change—the jump/drop—is observed, without the maximum (Figure 1b). However, in our previous study of (1−x)NBT-xCT solid solutions, where the measurements have covered a wider temperature range, we have observed a diffused hump in ε′(T) dependence around 450 °C, which earlier we have interpreted as surplus of this diffused part with a maximum at T_c_ [10] (see the additional graph in Figure 1b). (It should be noted that it was not possible to achieve high temperatures, reaching 450 °C, in the current study, due to technical limitations of the wide-frequency range equipment. Therefore, our previous work is mentioned in this context.)

For both NBT and 0.95NBT-0.05CT, the characteristic frequency-independent jump on heating and drop on cooling mark the region of pronounced temperature hysteresis, which is slightly wider than the jump and drop themselves. Particularly, in 0.95NBT-0.05CT, this hysteresis appears between 270 °C and 203 °C, while in NBT, it is observed just in the temperature range between 320 °C and 230 °C. Further, we focus mostly on this hysteresis region, giving a significant characterization to the studied samples. While the diffused part of the peak might have just a subordinate meaning, as it will be discussed below.

**Figure 2:**
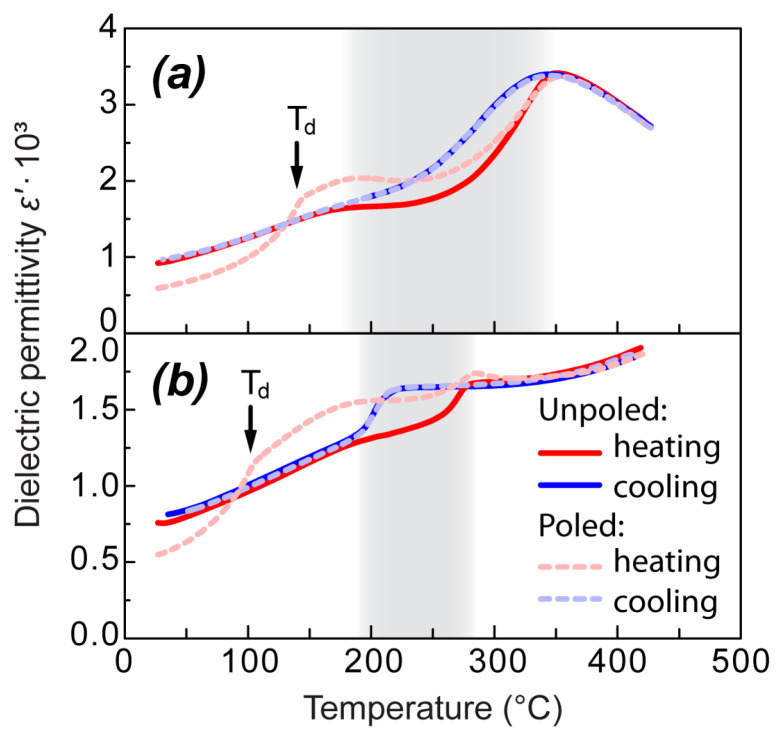
Temperature dependences of the real part of dielectric permittivity ε′(T) for the unpoled and poled NBT (**a**) and 0.95NBT-0.05CT (**b**) samples upon heating and cooling at a particular frequency of 860 Hz. The temperature hysteresis range is shaded grey. Rather low frequency is chosen in order to clearly demonstrate the ε′(T) shoulder which is more expressed in direction of low frequencies.

No thermal hysteresis was observed for the ε′(T) shoulder in the case of the unpoled samples. However, unambiguous differences in its behavior were observed upon heating of the poled samples (Figure 2). In this case, a well-expressed frequency-independent sharp increase in ε′(T) was observed at the depolarization temperature T_d_—141 °C for NBT and 103 °C for 0.95NBT-0.05CT. Taking into account that T_d_ is located in the low-temperature side of the shoulder, poling did not destroy the part of the shoulder located above T_d_. On the contrary, the shoulder after poling became much more expressed for both studied compositions, especially for 0.95NBT-0.05CT.

Mutual comparison of temperature dependences of dielectric permittivity ε′(T) on heating and on cooling of the unpoled and poled samples allows us to extract the following features of them for both NBT and 0.95NBT-0.05CT (in Figure 2, this comparison is illustrated at one chosen frequency—860 Hz):

1. In the case of the unpoled samples:
  ε′(T) has temperature hysteresis only in the temperature range marked by the jump and drop on heating and on cooling, respectively; outside this range, ε′(T) does not depend on the direction of temperature change and the heating and cooling curves coincide;
2. When the unpoled and poled samples are compared:
  - The heating curves coincide for the poled and unpoled samples only above the thermal hysteresis region, whereas in the temperature range below it, they differ significantly: (1) From room temperature up to the depolarization temperature T_d_, ε′(T) values in the poled sample are lower in comparison with the unpoled sample; (2) after the sharp increase at T_d_ up to the thermal hysteresis region, ε′(T) values in the poled sample are higher than in the unpoled sample;
  - The cooling curves for the poled and unpoled samples coincide completely. This confirms that heating above the temperature region of thermal hysteresis erases influence of poling on dielectric permittivity.

**Figure 3:**
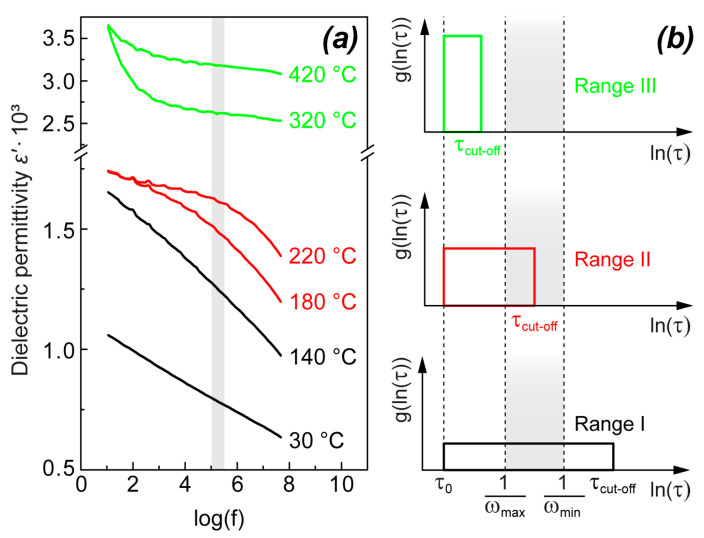
(**a**) Dielectric permittivity as a function of frequency logarithm ε′(log(f)) at several temperatures, representing three characteristic temperature ranges for unpoled NBT as examples: Range I (30 and 140 °C), where ε′(log(f)) is linear in the whole frequency range; Range II (180 and 220 °C), where a diffused transition between linear and practically frequency-independent ε′(log(f)) occurs; Range III (320 and 420 °C), where ε′(log(f)) is practically frequency-independent in the whole frequency range, but it has a significant contribution from the high-temperature conductivity at low frequencies. (**b**) Distribution functions of relaxation times g(lnτ) in three temperature ranges directly corresponding to those mentioned in context of the ε′(log(f)) measurements: High-temperature range (Range III) where the whole distribution function is outside the measurement frequency range; middle-temperature range (Range II) where the inverse maximal relaxation time 1/τ_cut-off_ is within the measured frequency range; and low-temperature range (Range I) where 1/τ_cut-off_ is below the measured frequency range. 1/ω_max_ and 1/ω_min_ mark the measurement frequency range on the lnτ scale.

The real part of dielectric permittivity as a function of frequency logarithm ε′(log(f)) also has similar behavior for both NBT and 0.95NBT-0.05CT and possesses features characteristic for relaxor ferroelectrics. This can be well seen from the examples of ε′(log(f)) for the unpoled NBT, depicted in Figure 3a at several temperatures, which represent three temperature ranges with different characteristic frequency dependences:

1. Range I (below the frequency-dependent shoulder)—with linear ε′(log(f)) in the whole measurement frequency range;
2. Range II (in the temperature region of the frequency-dependent shoulder)—with linear ε′(log(f)) at high frequencies and diffused non-linear transition to the practically frequency-independent ε′(log(f)) at low frequencies;
3. Range III (above the frequency-dependent shoulder)—practically frequency-independent ε′(log(f)) in the whole frequency range, supplemented by a contribution from the high-temperature conductivity, giving significant rise in ε′ at low frequencies.

**Figure 4:**
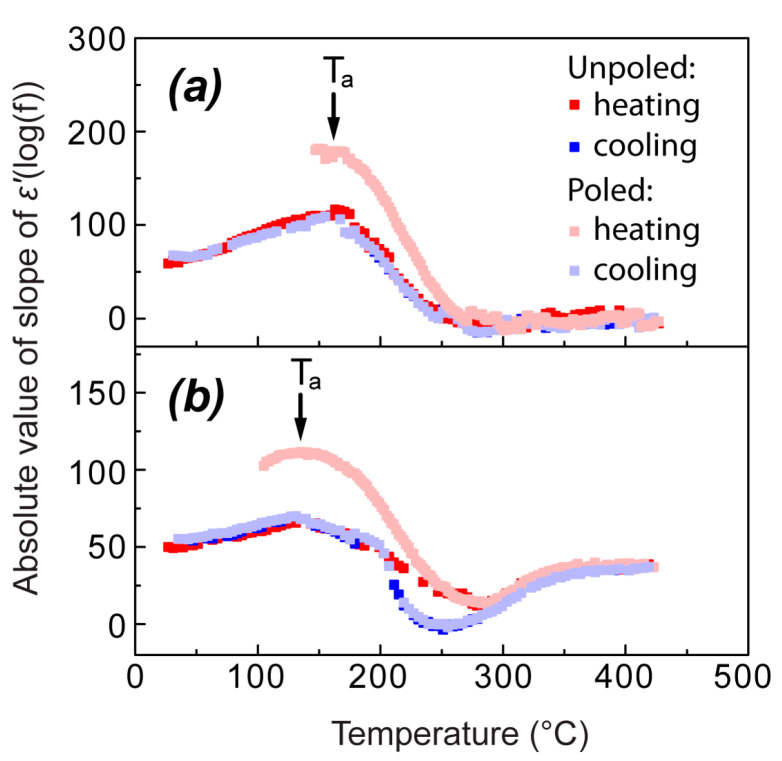
Temperature dependences of the absolute value of slope of ε′(log(f)), calculated for the frequency range 150–370 kHz, for the unpoled and poled NBT (**a**) and 0.95NBT-0.05CT (**b**) samples upon heating and cooling. The particular frequency range is chosen in such a way to avoid the contribution from the low-frequency dispersion, appearing at high temperatures, as much as possible, on the one hand, and the electromechanical resonance range, observed for the poled samples, on the other hand.

Temperature dependences of the absolute values of slope of ε′(log(f)) (further denoted simply as “slope of ε′(log(f))” for brevity) are determined for a particular frequency range of 150–370 kHz upon heating and cooling of the unpoled and poled NBT and 0.95NBT-0.05CT and are shown in Figure 4. This frequency “window” is shaded grey in Figure 3a. The choice of the frequency range here was performed in such a way to avoid the electromechanical resonance range, observed for the poled samples in MHz region, as well as to reduce the influence of conductivity, which gives a significant contribution to dielectric permittivity in the high-temperature range at low frequencies.

Upon heating of the unpoled samples, the ε′(log(f)) slope increases, reaching a maximum at a temperature Ta. It should be noted that Ta depends on the frequency range where the slope is determined. In the chosen frequency “window”, Ta for NBT is 160 °C (Figure 4a), while, for 0.95NBT-0.05CT, it is 130 °C (Figure 4b). At lower temperatures (Range I, according to the notation above), the increasing of the ε′(log(f)) slope reflects the fact that the linear dependence of ε′(log(f)) becomes steeper. In the case of NBT, it can be well seen for the temperatures 30 °C and 140 °C, shown as examples, in Figure 3. As it follows from Figure 3a, the practically frequency-independent part of ε′(log(f)) appears from the low-frequency side upon increasing temperature. We start to observe the decreasing of the ε′(log(f)) slope in Figure 4, marking Ta, as soon as the diffused transition to the region of the frequency-independent part of ε′(log(f)) approaches the considered frequency “window”, starting to give contribution to the calculated slope. It can be seen also qualitatively for 180 °C and 220 °C, shown as examples, in Figure 3a. In the case of NBT, this continues until the practically frequency-independent part of ε′(log(f)) completely determines ε′ in the whole considered frequency “window”. At this point, the determined slope value becomes equal to 0 and does not change upon further increasing temperature (Figure 4a). The transition between the linear part and the frequency-independent part of ε′(log(f)) is very diffused, as it can be seen qualitatively in Figure 3a for the temperatures 180 °C and 220 °C and as it is reflected in Figure 4a by the wide temperature range between Ta and the temperature where ε′(log(f)) becomes equal to 0. In the case of NBT, this transition extends in approximately a 100 °C range.

It should be also noted that, at high temperatures, dielectric permittivity experiences increase in the low-frequency region, which can be seen, for example, for the curve at 420 °C in Figure 3a. This low-frequency dispersion is attributed to the characteristic growth of conductivity at high temperatures, which starts to give contributions to ε′(log(f)). The range of this dispersion is outside the frequency “window” where we have quantitatively determined the slope. Therefore, its traces are not visible in Figure 4a.

In the case of unpoled 0.95NBT-0.05CT, the temperature range with almost frequency-independent ε′(log(f)), and thus ε′(log(f)) slope equal to 0, could not be distinguished. On the contrary, upon further increasing of temperature, above the range of continuous decreasing of ε′(log(f)) slope, the value of the slope increases again (Figure 4b). This behavior is obviously related to the significantly more expressed growth of conductivity at high temperatures compared to NBT, giving rise to the low-frequency dispersion and its contribution to the total ε′(log(f)) dependence, including the considered frequency “window”.

For the unpoled samples, the temperature dependences of the ε′(log(f)) slope in the cooling runs are very similar to the heating runs in the whole temperature range outside the thermal hysteresis region, where clear differences are observed in the case of 0.95NBT-0.05CT (Figure 4b). In the case of NBT, the thermal hysteresis of ε′(T) is not so expressed. This could be the reason why the heating and cooling curves of the ε′(log(f)) slope seem to coincide practically in all considered temperature ranges (Figure 4a).

Taking into account that, when poled, both NBT and 0.95NBT-0.05CT are in ferroelectric state, it is reasonable to consider the ε′(log(f)) slope of the poled samples only above T_d_—in the context of relaxor behavior. However, also in a certain temperature region above T_d_, the slope of ε′(log(f)), determined in the 150–370 kHz frequency “window”, significantly differs from the slope of the unpoled samples (Figure 4a,b). The observed differences could have been expected, as dielectric permittivity in the part of the shoulder remaining above T_d_ is significantly higher in comparison with the unpoled state.

Cooling curves of the ε′(log(f)) slope both for the poled NBT and 0.95NBT-0.05CT completely coincide with the cooling curves of the unpoled samples (Figure 4a,b). This, once again, confirms that heating above Tm completely erases the signs of poled state.

## 4. Discussion

The obtained results allow us to discuss behavior of dielectric properties which could be common for a large number of NBT-based compositions. They refer to both dependence on temperature and frequency. Concerning the temperature dependence of the real part of dielectric permittivity ε′(T) at any particular frequency, for now, we will address two characteristic properties observed for NBT and 0.95NBT-0.05CT:

Thermal hysteresis of ε′(T) for the unpoled samples of both compositions is limited to the temperature range between the dielectric permittivity jump on heating and drop on cooling and does not influence ε′(T) outside it. This allows us to consider dielectric permittivity behavior inside and outside this range separately.

As it is indicated by comparison of ε′(T) for the poled and unpoled samples, especially in the case of 0.95NBT-0.05CT, the ε′(T) shoulder and depolarization are two separate features on the temperature scale. In the poled state, dielectric permittivity in the region of the shoulder at temperatures above T_d_ is significantly higher.

Three ε′(lnf) dispersion features can be distinguished within the measured temperature–frequency range:

1. Quasilinear ε′(lnf) dispersion at low temperatures, observed in the whole frequency range, which transforms into almost frequency-independent ε′ above the ε′(T) shoulder;
2. Dielectric dispersion which appears at high temperatures and is especially expressed in the low-frequency range. This dispersion is obviously related to the increased dc conductivity at high temperatures.
3. In the poled samples, diffused resonance-type dispersion, observed at temperatures below T_d_ in the range of several MHz, is obviously created by the electromechanical resonance.

In our further discussion, we will focus on the quasilinear ε′(lnf) dependence (the first ε′(lnf) dispersion feature), which is apparently related to relaxor behavior in the studied compositions. Similar dispersion was observed in NBT single crystals [34] and 0.7NBT-0.3Sr_0.7_Bi_0.2_TiO_3_ ceramics, as well [46]. Like in other relaxor ferroelectrics, such dielectric dispersion can be explained by flipping of dipole moments of PNRs due to thermal agitation. Introduction of distribution of relaxation times g(lnτ) allows us to express the dielectric dispersion as follows [50]:

(1) $$\varepsilon^{\prime }(\omega )=\varepsilon_{\infty }+\Delta \varepsilon \int_{-\infty }^{\infty }\frac{g(\ln\tau )d(\ln\tau )}{1+\omega^{2}\tau^{2}}$$

where dielectric strength Δε = ε_s_ − ε_∞_ is the difference between ε′ in the low-frequency side (ε_s_) and the high-frequency side (ε_∞_) of the dielectric dispersion, and ω is angular frequency, equal to 2πf.

Macutkevic et al. have attempted to construct the shape of the arbitrary distribution function g(lnτ) presented in Equation (1) for particular relaxor ferroelectrics using the experimentally obtained data of dielectric dispersion and applying the Tikhonov regularization method [51]. However, the main features of dielectric dispersion in relaxor ferroelectrics can be reflected even assuming a much more simple shape of distribution function—as follows:

(2) $$g(\ln(\tau ))=D=\frac{1}{\ln(\tau_{cut-off}/\tau_{0})},\;\mathrm{if}\;\tau_{0}<\tau <\tau_{\mathrm{cut-off}},\;g(\ln(\tau ))=0,\;\mathrm{if}\;\tau <\tau_{0}\;\mathrm{or}\;\tau >\tau_{\mathrm{cut-off}}$$

If these assumptions are made and if, additionally, 1/(1 + ω^2^τ^2^) is approximated by a step function with value 0, if ωτ > 1, and with value 1, if ωτ < 1 (this approximation influences ε′(lnω) only close to 1/τ_cut-off_ and 1/τ_0_, making this dependence smoother), a linear dependence of ε′(lnω) is obtained within the frequency range 1/τ_cut-off_ < ω < 1/τ_0_:

(3) $$\varepsilon^{\prime }(\omega )=\varepsilon_{\infty }+\Delta \varepsilon ,\;\mathrm{if}\;\omega <1/\tau_{\text{cut-off}},\;\varepsilon^{\prime }(\omega )=\varepsilon_{\infty }+\frac{\Delta \varepsilon }{\ln\tau_{cut-off}-\ln\tau_{0}}\left(-\ln\omega -\ln\tau_{0}\right),\;\mathrm{if}\;1/\tau_{\text{cut-off}}<\omega <1/\tau_{0}\;\varepsilon^{\prime }(\omega )=\varepsilon_{\infty },\;\mathrm{if}\;\omega >1/\tau .$$

Remarkably, the linear dependence of ε′(lnω), together with the frequency-independent imaginary part of dielectric permittivity ε″, observed in relaxor ferroelectrics at temperatures significantly below T_m_, naturally follows from this concept if τ_cut-off_ >> 1/ω_min_. Meanwhile, unsurpassable difficulties are met when trying to describe these experimental observations by the frequently used relaxation functions, such as the Cole–Cole and the Havriliak–Negami relations [51].

The simplified dependence in Equation (3) corresponds to the main characteristics of the experimentally measured ε′(lnω), represented by the examples at several temperatures in Figure 3a. In the whole studied frequency and temperature range, ω_max_ < 1/τ_0_. In the temperature region where ω_max_ > 1/τ_cut-off_ and ω_min_ < 1/τ_cut-off_, ε′(lnω) consists of two parts with a diffused transfer between them: (1) the frequency-independent part at frequencies ω < 1/τ_cut-off_ and (2) the linearly decreasing part, following Equation (2), at frequencies ω > 1/τ_cut-off_ (the curve at 220 °C and to some extent also the curve at 180 °C, as examples, in Figure 3a). In the temperature region where ω_max_ < 1/τ_cut-off_, dielectric permittivity is frequency-independent in the whole measured frequency range if the contribution from conductivity is ignored (the curves at 320 and 420 °C, as examples, in Figure 3a). Such an approach is supported by the behavior of dielectric permittivity above the temperature range of the shoulder in NBT single crystals, where the frequency dependence of dielectric permittivity vanishes below MHz range [21,34], whereas in the temperature range in which ω_min_ >> 1/τ_cut-off_, the linear dependence of ε′(lnω) is observed in the entire frequency range (the curves at 30 and 140 °C, as examples, in Figure 3a).

It is widely assumed that, in relaxor ferroelectrics, temperature dependence of the maximal relaxation time τ_cut-off_(T) follows the Vogel–Fulcher law [52] up to complete freezing at a certain temperature T_f_, while τ_0_ is only weakly dependent on temperature. Just increasing τ_cut-off_ in the direction of low temperatures determines the shift in the ε′(T) maximum towards the low temperatures upon reducing the measurement frequency. In the temperature range above T_m_(ω_min_), dielectric permittivity at lower frequencies is outside the dispersion range and is equal to ε_s_ according to Equation (3). In relaxor ferroelectrics, ε_s_ increases at low temperatures. Nevertheless, the substantial widening of the distribution function g(lnτ) leads to the reduction in the ε′(lnω) slope [53], which depends on τ_cut-off_ and Δε according to Equation (3). In the temperature range below T_m_(ω_min_), where the frequency-independent part of dielectric permittivity at low frequencies is no longer observed in the measured frequency range, ε_s_(T) cannot be directly determined. On the other hand, increasing ε_s_ upon cooling also in the temperature region between T_f_ and T_m_(ω_min_) seems to be quite natural. Moreover, as per some estimates [53], due to the extremely rapid widening of g(lnτ) upon approaching T_f_ according to the Vogel–Fulcher law, ε_s_(T) must exhibit significant growth towards lower temperatures to make it possible to fit the experimentally observed ε′(T, ω) below T_m_(ω) under Equation (3). Otherwise, the slope of ε′(T) below T_m_ at low frequencies is much steeper compared to the experimentally observed slope [53]. However, the dielectric permittivity maximum at T_c_ in NBT-based compositions in most cases does not possess a clear shift with measurement frequency, characteristic of the conventional relaxor ferroelectrics. Therefore, the reduction in ε′(T) below T_c_ in the low-temperature direction cannot be explained based on the above considerations. This difference is highlighted by the findings in two reports of dielectric dispersion in NBT-based compositions: NBT single crystals [45] and 0.7NBT-0.3Sr_0.7_Bi_0.2_TiO_3_ ceramics [46], mentioned in Section 1. In both of these studies, it was concluded that dielectric strength Δε is responsible for the maximum at T_c_ in the temperature dependence of dielectric permittivity.

In the present research, due to the highly pronounced contribution of conductivity to dielectric permittivity at low frequencies (see the curves at 320 and 420 °C in Figure 3a), direct determination of εs, in the way it was succeeded in (1−x)NBT-xSr_0.7_Bi_0.2_TiO_3_, is not possible. However, we assume that presence of the maximum in ε_s_(T) is a common feature for all NBT-based compositions possessing the considered temperature dependence of dielectric permittivity: exhibiting the maximum at T_c_, which does not shift in dependence on measuring frequency, and the frequency-dependent shoulder below this maximum. In the present study, the absence of dependence on frequency for dielectric permittivity in NBT in a range of hundreds of kHz (Figure 4a), extending from the high-temperature range to some temperature region below T_c_, can also be attributed to the remaining part of the frequency-independent feature. Therefore, it can be considered as a signature of reduction in ε_s_ upon decreasing of temperature below T_c_.

Taking into account that the observed behavior of ε_s_ below T_c_ cannot be related to the relaxor behavior, we have to consider other reasons explaining it. In 0.95NBT-0.05CT, the temperature of the jump/drop of ε′(T, ω) below T_c_ and its thermal hysteresis coincide with the temperature and thermal hysteresis of the first-order structural phase transition between two nonpolar phases—orthorhombic and tetragonal [10]. Therefore, in this case, treating this jump/drop as a result of a phase transition between phases with different Δε values seems to be appropriate. As it was pointed out in our earlier publication [10], temperature dependence of ε′(T, ω) in NBT is similar to that of 0.95NBT-0.05CT—it also has pronounced thermal hysteresis in a certain temperature range above the shoulder, containing the jump/drop (Figure 1a). In the case of NBT, the link to a structural phase transition is not easy to establish because the phase transition is much more diffused [4,19,20]. However, Ge et al. [21] have observed that the temperature of the phase transition to the tetragonal phase upon heating corresponds to the jump in ε′(T, ω). At the same time, the thermal hysteresis between the jump and the drop of ε′(T, ω) coincides with that of elastic compliance [47], which indirectly confirms the relation of the jump/drop of ε′(T, ω) to the phase transition.

The discussed phase transition changes only ε_s_ (or Δε) above the frequency-dependent shoulder, leaving the dielectric relaxation untouched. Taking this into account, we can imagine that the peak in ε′(T, ω) could be eliminated by reduction in ε_s_ or Δε, leading to the pure relaxor behavior, not influenced by this phase transition. Such compensation can be achieved at an appropriate value of a coefficient k introduced to reflect the ratio between the reduced value of ε_s_ and the real one $k=\frac{\varepsilon_{s,\;reduced}}{\varepsilon_{s,real}}$. At the same time, k is also the ratio between the corrected and the measured ε′ at any frequency within the relaxation range. This easily follows from Equation (3).

**Figure 5:**
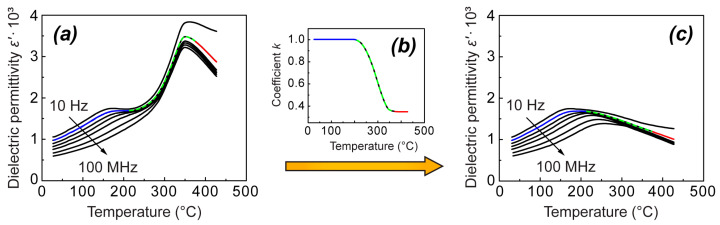
ε′(T) at different frequencies for the unpoled NBT on heating (**a**), correction to static dielectric permittivity ε_s_ due to nonferroelectric phase transition, represented by multiplication coefficient k (**b**) and constructed ε′(T) without influence of phase transition (**c**).

To determine the coefficient k as a function of temperature from our measurements, we have considered temperature dependence of dielectric permittivity at one particular chosen frequency—360 Hz. The low-temperature segment of the ε′(T), including the shoulder, was left unchanged (the blue segment in Figure 5a,c)—multiplication coefficient k here is equal to 1 (Figure 5b). The high-temperature segment of the ε′(T) curve starting slightly above T_c_ (the red segment in Figure 5a) was multiplied by a constant coefficient k, chosen in such a way that this segment is shifted downwards to visually form a smooth diffused peak together with the low-temperature segment (the red segment together with the blue segment in Figure 5c). Further, the missing part of the constructed ε′(T) curve between the blue and the red segments was interpolated (green dashed segment in Figure 5c). The coefficient k in this range was determined as the ratio between the values on the interpolated curve and the experimental values at 360 Hz. Thus, determined temperature dependence of the coefficient k at 360 Hz is shown in Figure 5b. Further, the determined values of k were used to calculate the transformed dielectric permittivity dependences on temperature (Figure 5c) from the experimentally measured curves (Figure 5a) at all other frequencies by multiplying the experimental data at each particular frequency by the values of k determined at 360 Hz (Figure 5b).

Figure 5c represents the results obtained by this approach for the heating curves of the unpoled NBT. Indeed, in such a way, the experimental curves are transformed into the smooth ε′(T, ω) dependences, characteristic of relaxor ferroelectrics, in the whole frequency range. We assume that such dependences describe the dielectric dispersion in NBT in the absence of the phase transition. However, it should be noted that the temperature dependences of dielectric permittivity obtained in this way are suitable for illustrative purposes only—the choice of k is, to some extent, arbitrary, as the only criterion to derive it is obtaining a smooth behavior or ε′(T) between the temperature range of the frequency-dependent shoulder and the temperature range above the thermal hysteresis region.

**Figure 6:**
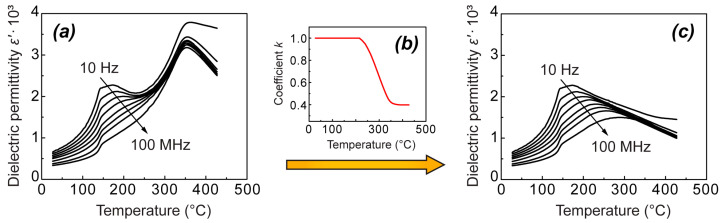
ε′(T) at different frequencies for the poled NBT on heating (**a**), correction to static dielectric permittivity ε_s_ due to non-ferroelectric phase transition, represented by multiplication coefficient k (**b**) and constructed ε′(T) without influence of phase transition (**c**).

Similar results were obtained also after applying the same approach to the heating curves of the poled NBT sample, with the difference that the maximal values of the transformed ε′(T) are significantly higher (see Figure 6). Another difference is the break in ε′(T) below the maximum, which is related to the depolarization. In the framework of the proposed concept, the depolarization temperature can be considered the temperature of the irreversible phase transition from the ferroelectric state to the relaxor state upon heating of a previously poled traditional relaxor ferroelectric, possessing solely frequency-dependent maximum of ε′(T) [50,54,55]. As it is known for relaxor ferroelectrics, the depolarization temperature T_d_ can be located far below the frequency-dependent ε′(T) maximum, which is known not to be related to any phase transition. In addition to the arguments mentioned in Section 1, the discussed similarities between NBT-based compositions and traditional relaxor ferroelectrics support the hypothesis that the frequency-dependent ε′(T) shoulder in NBT-based compositions does not correspond to the depolarization temperature T_d_.

At first glance, similar treatment of dielectric permittivity in 0.95NBT-0.05CT could be even simpler, taking into account that the jump/drop is less diffused and dielectric permittivity only weakly depends on temperature at high frequencies above this jump/drop. However, a much larger contribution of conductivity creates strong frequency dependence of dielectric permittivity in high temperature region. For this reason, the simple approach, used for elimination of jump contribution in NBT, is not applicable in this case. However, from our point of view it does not mean that the presented interpretation of temperature dependence of dielectric permittivity fails in this case.

Considering common properties of relaxor ferroelectrics and the studied NBT and 0.95NBT-0.05CT compositions, we have to stress also a difference between them: in NBT and 0.95NBT-0.05CT, poling leads to appearance of significantly more pronounced relaxor behavior above T_d_ compared to the unpoled samples, while, in traditional relaxor ferroelectrics, this does not happen. This difference could be related to the fact that, in NBT and 0.95NBT-0.05CT, the concentration of PNRs remaining after destruction of the poled state above T_d_ is higher than the concentration of PNRs in the unpoled state in the same temperature range. In our previous study, comparison of the X-ray diffraction pattern obtained for the poled and the unpoled 0.95NBT-0.05CT sample in the temperature region above T_d_ revealed clear differences. However, we were not able to relate these differences to the increased concentration of the rhombohedral phase in the poled samples above T_d_ [10].

In the framework of the proposed interpretation, the characteristic dielectric permittivity maximum of NBT at T_c_ could have no independent meaning but is rather a side effect of the diffused jump of Δε at the phase transition between two nonferroelectric phases. This maximum was interpreted in the same way in studies of NBT-PbTiO_3_ [56], where it is located clearly above the structural phase transition. We also came to a similar conclusion in studies of NBT-BaTiO_3_ solid solutions in the tetragonal side from the morphotropic phase boundary [57]. Since the frequency-dependent ε′(T, ω) shoulder reflects the relaxor-characteristic behavior, it is not indicative of a phase transition but just reflects temperature dependence of the relaxation time. The values of ε′(T, ω) in its vicinity are too high to be related to pure nonferroelectric phases. However, we have to take into account the relaxor nature of the dielectric properties. First of all, it concerns the phase in the temperature region below the jump/drop of ε′(T). Presence of PNRs in this phase is usually assumed, and the high values of dielectric permittivity could be related to the contribution of these PNRs, like in traditional relaxor ferroelectrics. Apparently, preserving of PNRs should be assumed also after the transfer into the high-temperature phase. At first glance, considering flipping of dipole moments of PNRs, it could be quite natural to relate the jump/drop of ε′(T) with the change in crystalline structure inside PNRs, just as it was assumed for NBT-based compositions by Jo et al. in their study [42]. Thus, the jump/drop of the unit cell dipole moment values is a consequence of this change. However, it is not clear why the dipole moments of PNRs should have higher values in the high-temperature phase. Taking into account that the P4bm phase in NBT is only weakly polar [4], rather the opposite could be assumed—PNRs in the low-temperature phase possessing the R3c symmetry should have larger dipole moments than the ones in the high-temperature P4bm phase. In addition, in our previous studies of second harmonic generation (SHG), monotonous reducing of SHG signal intensity was observed upon increasing temperature both below and above the phase transition temperature either in NBT or in 0.95NBT-0.05CT, which contradicts with such a change in the dipole moments [47].

Based on our observation regarding SHG, we have suggested that the reason for the jump/drop of dielectric permittivity in the low-temperature side of T_c_ could be the change in interaction between PNRs and the surrounding matrix due to the symmetry change in the matrix [47]. This idea could more correspond to the “breathing” model of PNRs [58] than to the flipping model. It also allows assuming that symmetry of PNRs does not change at the phase transition, avoiding the above-mentioned contradiction. The change in Δε in this case can be explained by differences in surface tension of PNR boundaries in both matrix phases. These differences result in variations in bending of PNR boundaries by the electric field and, as a consequence, in changes in PNR volume and total dipole moment. It should be pointed out that the characteristic logarithmic frequency dependence of dielectric permittivity (Figure 3a), which can be described by Equation (3), also corresponds to the “breathing” model [44]. In this model, the distinction between the cases of linear ε′(lnω) dependence and the frequency-independent dielectric permittivity (quasistatic regime) depends on the relation between the size of PNRs and the pinning free distance. If PNRs are of R3c symmetry, their existence above the nonferroelectric phase transition does not contradict with the assumed coexistence of P4bm and R3c in this temperature region [4,19].

It is likely that the above-described concept is applicable also for other NBT-based compositions, where ε′(T, f) behavior characteristic of NBT is observed. Moreover, it could be even extended beyond such behavior, assuming that the sequence of the non-ferroelectric phase transition and the temperature region where the dielectric dispersion appears on temperature axis could differ depending on the composition and, presumably, even depending on variations in the producing route of the material. This could explain the differences in ε′(T, f) between NBT, on the one hand, and behavior in (1−x)NBT-xPbZrO_3_ solid solutions, on the other hand [22]. Unlike the case discussed in this study, the maximum dielectric permittivity in these compositions shifts with frequency, closely aligning with traditional relaxor ferroelectrics. Apparently, the upper temperature limit, at which the distribution of the relaxation times is significant, in the case of (1−x)NBT-xPbZrO_3_ and Bi-over stoichiometric NBT, extends above the temperature of the structural phase transition, to a large extent hiding the features of it. Similar superimposion of the structural phase transition to the maximum in the temperature dependence of dielectric permittivity characteristic for relaxor ferroelectrics can exist also in (1−x − y)NBT-xSr_0.7_Bi_0.2_TiO_3_-yPbTiO_3_ solid solutions [58]. The specific thermal hysteresis of this maximum can be related to thermal hysteresis of the structural phase transition, which extends in the temperature region of the dielectric dispersion.

In the present study, elimination of the jump/drop contribution through calculations leads to the transformed ε′(T, f) dependences with pure relaxor-characteristic behavior, whereas in the case of (1−x)NBT-xSr_0.7_Bi_0.2_TiO_3_, it can be assumed that such elimination occurs in reality upon increasing of Sr_0.7_Bi_0.2_TiO_3_ content—the maximum at T_c_ continuously reduces upon increasing of x until it finally disappears, leaving just the pure relaxor-characteristic behavior [27,46]. Apparently, rise of Sr_0.7_Bi_0.2_TiO_3_ concentration leads to continuous weakening of features of the structural phase transition and its influence on PNRs until they disappear. The opposite scenario is observed in (1−x)NBT-xPbTiO_3_ solid solutions where an already small amount of PbTiO_3_ completely suppresses the shoulder. At the same time, the well-expressed jump in ε′(T) dependence upon heating corresponds to a convincingly detected structural phase transition between the rhombohedral and the high-temperature phase with presumably tetragonal phase [56]. While, as it was noted above, further increasing ε′(T) up to the maximum is interpreted as a “diffused region”, which also belongs to a ferroelectric–tetragonal phase transition but is already inside the structure of the high-temperature tetragonal phase. Behavior of ε′(T), observed in both cases, confirms mutual independence of the jump and the shoulder, observed in NBT-based compositions, as well as correspondence of the jump in ε′(T) to the structural phase transition.

Thus, the presented concept, implementing mutually independent non-ferroelectric phase transition, on the one hand, and dielectric dispersion characteristic for relaxor ferroelectrics, on the other hand, allows explaining the specific behavior of dielectric properties of NBT-based compositions. The sequence of these two mechanisms on the temperature axis can vary, resulting in significant differences in the temperature dependences of dielectric permittivity, which are experimentally observed. The proposed concept seems to be appropriate also with respect to phase diagrams of these compositions available in the literature.

## 5. Conclusions

New interpretation of temperature dependence of dielectric permittivity in NBT-based compositions is proposed, based on the investigation of dielectric properties in two compositions: NBT and 0.95NBT-0.05CaTiO_3_. The temperature dependence of dielectric permittivity in both compositions is described by two contributions: one from the structural phase transition between two non-ferroelectric phases, and one from pure relaxor behavior, which is performed in this way for the first time. The steep change (jump/drop) in the temperature dependence of dielectric permittivity above the frequency-dependent shoulder, which possesses well-expressed thermal hysteresis, is explained as the result of the first-order phase transition between two non-ferroelectric phases, while PNRs are preserved both below and above the transition. The latter explains the high values of dielectric permittivity in these phases. The jump/drop, characterizing dielectric properties by relaxation mechanism, is reflected solely in change in dielectric strength. The so-called “breathing” model is applied. The difference in dielectric strength in both non-ferroelectric phases, in the framework of this concept, is explained by the change in the interaction between PNRs and matrix in these phases.

If the contribution of the phase transition between two non-ferroelectric phases is excluded from the dielectric strength, behavior of dielectric permittivity in the studied compositions corresponds well to the one characteristic of classical relaxor ferroelectrics. First of all, the frequency-dependent shoulder in the temperature dependence of dielectric permittivity transforms into a frequency-dependent maximum, corresponding to the general concept of relaxor ferroelectrics. Secondly, steep change in ε′(T) at heating of the poled samples below the shoulder corresponds to the phase transition from the ferroelectric to relaxor state.

## Data Availability

The original contributions presented in this study are included in the article. Further inquiries can be directed to the corresponding author.
